# Predictors of consciousness improvement in patients with hypoglycemic encephalopathy

**DOI:** 10.3389/fendo.2022.956367

**Published:** 2022-08-16

**Authors:** Yu Eun Lee, Eun Ja Lee, Seung Eun Lee, Jinkyeong Park

**Affiliations:** ^1^ Department of Internal Medicine, Dongguk University Ilsan Hospital, Goyang, South Korea; ^2^ Department of Radiology, Dongguk University Ilsan Hospital, Goyang, South Korea; ^3^ Department of Pulmonary, Allergy and Critical Care Medicine, Kyung Hee University Hospital at Gangdong, College of Medicine, Kyung Hee University, Seoul, South Korea

**Keywords:** hypoglycemia, diabetes mellitus, magnetic resonance imaging, metabolic brain diseases, encephalopathy, medical futility

## Abstract

**Aims:**

Hypoglycemic encephalopathy (HE) can cause long-lasting mental changes, disability, and even death. We aimed to investigate prognostic factors for HE and to determine when the treatment of HE becomes futile.

**Methods:**

We retrospectively evaluated the data of patients admitted for prolonged HE at Dongguk University Ilsan Hospital between December 2005 and July 2021. We assessed the Glasgow Outcome Scale (GOS) to assess functional outcome.

**Results:**

Forty-four patients were enrolled in the study. Thirty-two of these showed the improvement on GOS after treatment. Patients with improved consciousness had a shorter duration of hypoglycemia (1.6±1.4 vs. 7.8±15.0 hours, *p* = 0.04) and a lower incidence of brain lesions than those without improvements in consciousness (76.0% vs. 25.0%, *p* < 0.01). Patients whose lesions were detected in initial MRIs were 1.3 times less likely to recover consciousness after HE (odds ratios, 1.28; 95% CI, 1.09-1.52; *p* < 0.01). None of the patients recovered consciousness after 320 h. Maximum time spent to recover was 194 in patients without brain lesions and 319 in those with lesions.

**Conclusions:**

Hypoglycemic brain injury detected in initial MRIs predicted poorer HE prognosis. Nevertheless, treatment should be provided for at least for 14 days after admission.

## Introduction

Hypoglycemia is a significant complication of diabetes (1). Severe hypoglycemia has been reported in 7.1%-16.5% of patients with diabetes mellitus (DM) (2–5). The symptom and signs of hypoglycemia range from feeling like a shark to cognitive dysfunction and seizure. Mild hypoglycemia reduces the quality of life, while severe hypoglycemia can be life-threatening. Hypoglycemic encephalopathy (HE) is a condition in which patients become comatose or enter prolonged stupor despite normalization of blood glucose levels. This can cause long-lasting mental changes, disability, or even death (6).

The factors that determine whether HE leads to a persistent vegetative state, complete recovery, or anything in between remain unclear. The functional brain failure that can result from hypoglycemia is on a continuous spectrum of progressively increased risk of neuronal death at lower plasma glucose levels (7). Several mechanisms cause hypoglycemia-induced brain damage. These include interruption of blood glucose supply during a hypoglycemic episode and reperfusion injury from therapeutic glucose supply. Factors found to be related to the recovery of consciousness after a hypoglycemic episode include initial blood glucose levels, body temperature when hypoglycemic occurs, and brain lesion (6, 8–10). However, research has been limited to case studies and case involving a small number of patients, and results have varied from study to study (6, 10).

More studies on clinical factors that can predict recovery after HE are needed to avoid the futile treatment of patients with irreversible brain injury and ensure the provision of intensive treatment in patients with reversible brain injury. In this study, we aimed to investigate the prognostic factors in HE patients and evaluate the timing at which the treatment of HE becomes futile.

## Materials and methods

### Study population

We retrospectively evaluated the data of patients admitted to Dongguk University Ilsan Hospital with prolonged HE between December 1, 2005, and July 31, 2021. A diagnosis of prolonged hypoglycemic encephalopathy was considered in all patients >18 years old admitted with the following: (1) a score of fewer than 13 points on Glasgow Coma Scale (GCS). (2) hypoglycemic etiology of unconsciousness with at least one measurement of plasma glucose or capillary blood glucose of <54 mg/dL on presentation, and (3) no recovery of consciousness immediately after normalizing blood glucose levels. Patients with possible other causes of consciousness disorders and previous cognitive disorders were excluded. Possible causes of consciousness disorders include head trauma, cerebral infarction, cerebral hemorrhage, and metabolic encephalopathies such as sepsis. The Medical Research Ethics Review Committee of Dongguk University Ilsan Hospital approved this study (DUIH 2022-01-006).

### Data collection and operational definitions

To identify factors that can predict the improvement of consciousness from prolonged HE, we evaluated clinical variables on admission, during hospitalization, and on discharge. The duration of hypoglycemia was defined as the time between the point loss of consciousness was first verified and the start of treatment. Post-treatment hyperglycemia was defined as serum glucose greater than 200mg/dL for 2 hours post-treatment based on previous studies (10, 11).

Scores on the Glasgow Outcome Scale (GOS) were used to assess functional outcomes. Scores range from 1 to 5, with 5 indicating good recovery, 4 indicating slight disability that has not reached the previous functional level, 3 indicating disability without the capacity for independent daily living, 2 indicating absence of self-awareness and environmental awareness (vegetative state), and 1 indicating death. Improvement of consciousness was defined as an increase in GOS of 1 point or more at any time during hospitalization from that at the time of admission.

### MR imaging and EEG

Magnetic resonance imaging (MRI) results were retrospectively assessed by a a neuroradiologist (with 20 years of clinical experience). All MRIs were performed on a 1.5- T (Avanto, Siemens Healthcare) or 3-T (Skyra, Siemens Healthcare, Erlangen, Germany) units. Areas with restricted diffusion in typical locations of hypoglycemia (posterior limb of the internal capsule, centrum semiovale, corona radiata, cerebral cortex, hippocampus and basal ganglia) were defined as MRI finding suggestive of HE. Lesions were evaluated separately in each of the following four areas: (1) white matter (centrum semiovale, corona radiata, internal capsule, corpus callosum, and other white matter), (2) gray matter (frontal lobe, parietal lobe, temporal lobe, occipital lobe, insular lobe, and hippocampus), (3) deep gray matter (basal ganglia and thalamus), and (4) brain stem. Based on a previous study, we defined severe brain damage as high signal intensity in two or more lobs of gray matter (8). Lesions in the other areas except for gray matter or one lobe in gray matter in one lobe were considered non-severe brain damage.

Electroencephalography (EEG) findings were categorized as normal or abnormal. Background slow waves of 6–8 Hz are defined as normal, and background slow waves of ≤6 Hz are defined as abnormal.

### Statistical analysis

Clinical variables measured on admission were assessed for changes as factors potentially predicting improvement of consciousness in prolonged HE. Patients with improved GOS scores during hospitalization were compared to those without improvement. Student’s t-tests or Mann–Whitney U-tests were used to compare continuous variables, and chi-square tests or Fisher’s exact tests were used to compare categorical variables. Statistically significant variables were defined as predictive factors. For multivariate analysis, logistic regression and Cox’s proportional hazards model were used. A log-rank test was used to evaluate the improvement of consciousness in the presence or absence of lesions on MRI. Finally, survival curves were generated using the Kaplan–Meier method. All *p*-values were two-sided, and *p* < 0.05 was considered statistically significant. All analyses were performed using R (v. 4.0.3) (R Core Team, Vienna, Austria) software.

## Results

Of the 44 patients in our study, 38 (86.4%) had diabetes. After treatment, 72.7% (32/44) of patients showed improvement in GOS scores (**Table 1**). The mortality of this study was 11.4% (5/44). Among those with diabetes on treatment, the proportion of patients who suffered recurrent hypoglycemia was not different between groups (30% vs. 17.9%, p = 0.72). In addition, etiology of hypoglycemia, body temperature during hypoglycemia, and the proportion of post-treatment hyperglycemia were not different. The duration of the hypoglycemic episode was significantly shorter in patients showing GOS improvement after treatment than in those who did not (1.6 ± 1.4 vs. 7.8 ± 15.0, *p* = 0.04). MRI images were obtained on admission for 37 of the 44 patients. When brain lesions were identified on the initial MRIs, there was a lower incidence of improvement in GOS scores than in those without lesions (76.0% vs. 25.0%, *p* < 0.01). Of the 37 patients who underwent MRI on admission, 24 (64.9%) were followed up with further MRIs. Among those with lesions, patients whose consciousness improved showed greater improvement in lesions on MRI than those who did not (25% vs. 12.5%). On the other hand, worsening of the lesion was observed in three cases without improvement in consciousness and one case with improvement in consciousness.

**Table 1:** Baseline characteristics of patients with hypoglycemic encephalopathy.

	Patients without improving GOS	Patients with improving GOS	P-value
**Number of patients**	12	32	
**Age**	68.8 ± 12.0	69.8 ± 14.9	0.84
**Male (%)**	6 (50.0)	17 (53.1)	1.00
**BMI**	19.1 ± 3.2	21.4 ± 4.1	0.10
**Body temperature**	37.0 ± 0.9	36.5 ± 0.9	0.11
**Hospital LOS (days)**	54.6 ± 49.3	30.0 ± 57.3	0.20
**ICU LOS (days)**	31.8 ± 47.9	16. 3 ± 56.0	0.40
**Alcohol (%)**	3 (25.0)	10 (31.2)	0.97
**Diabetes**	10 (83.3)	28 (87.5)	1.00
Number of OHA classes*			0.34
** Less than 2**	6/9 (66.7)	11/27 (40.7)	
** 2 or above**	3/9 (33.3)	16/59.3 (59.3)	
Insulin*	5/9 (55.6)	14/27 (51.9)	1.00
Insulin and/or sulfonylurea*	9/9 (100)	27/27 (100)	
**Cause of hypoglycemia**			0.83
** Glucose-lowering therapy**	10 (90.9)	27 (87.1)	
** Alcohol**	0 (0)	1 (3.2)	
** Unknown**	1 (9.1)	3 (9.7)	
**Recurrent^#^**	3/10 (30.0)	5/28 (17.9)	0.72
**Duration of hypoglycemia**	7.8 ± 15.0	1.6 ± 1.4	0.04
**Initial glucose levels**	30.6 ± 15.0	33.8 ± 15.0	0.54
**Post-treatment hyperglycemia**	7 (77.8)	20 (66.7)	0.83
**GCS at ER**	7.8 ± 3.9	8.45 ± 4.0	0.61
**Seizure (%)**	2 (16.7)	12 (37.5)	0.34
**HbA1c**	6.9 ± 1.8	6.8 ± 2.1	0.86
**C-peptide**	2.1 ± 1.9	4.32 ± 5.1	0.21
**MRI (%) at initial**			<0.01
** Normal**	3 (25.0)	19 (76.0)	
** Abnormal,**	9 (75.0)	6 (26.9)	
** Non-severe**	3 (33.3)	4 (66.7)	
** Severe**	6 (66.7)	2 (33.3)	
**GOS at admission (%)**	3 (27.3)	6 (18.2)	0.83
** 2**	8 (75.0)	27 (81.2)	
** 3**	3 (25.0)	6 (18.8)	
**Follow-up MRI**	9/12 (75.0)	15/25 (60.0)	0.21
** Improved lesions in MRI**	1 (12.5)	4 (25.0)	
** No interval change in MRI**	5 (50.0)	10 (68.8)	
** Advanced lesions in MRI**	3 (37.5)	1 (6.2)	
**Best GOS (%)**			<0.01
** 2**	10 (81.8)	0 (0)	
** 3**	2 (18.2)	16 (50.0)	
** 4**	0 (0)	10 (31.2)	
** 5**	0 (0)	6 (18.8)	

BMI, body mass index; ER, emergency room, GCS, Glasgow Coma Scale; GOS, Glasgow Outcome Scale; ICU, intensive care unit; LOS, length of stay; MRI, magnetic resonance imaging.

*Estimated for those with diabetes on treatment and have information about medication.

^#^Estimated for those with diabetes on treatment.

**Figure 1:**
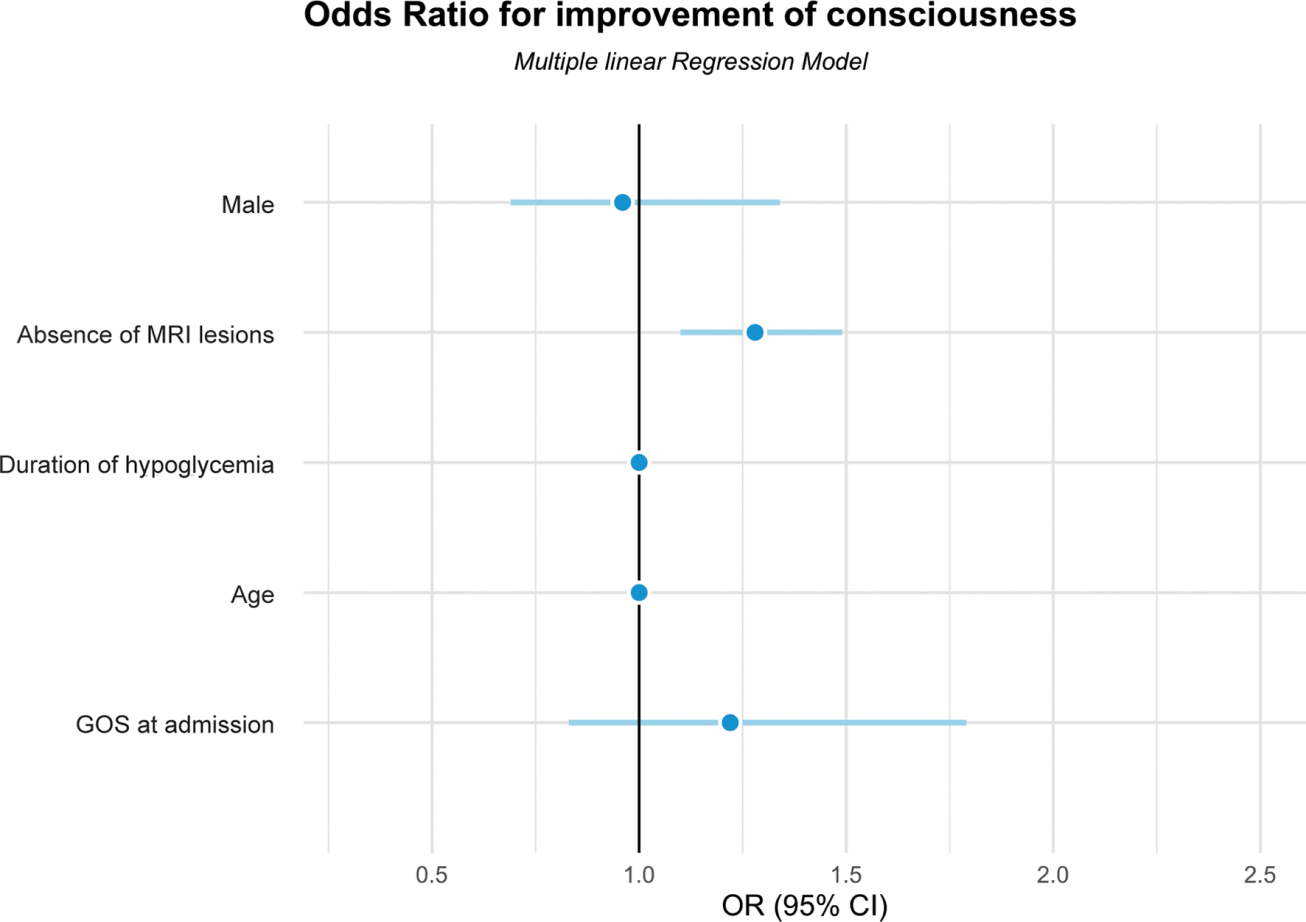
Odds ratios for improvement of consciousness in patients with hypoglycemic encephalopathy using the multiple linear regression model. GOS, Glasgow Outcome Scale; MRI, magnetic resonance imaging.

**Figure 1** shows the odds ratios (OR) for failure to recover consciousness after HE for each of the potential prognostic factors. Those without any brain lesions on their initial MRI were 1.3 times more likely to recover consciousness after HE (OR, 1.28; 95% CI, 1.10–1.49; p < 0.01). Those without severe brain damage on their initial MRI were 1.9 times more likely to recover consciousness after HE when compared to those with severe brain damage (OR, 1.87; 95% CI, 1. 29–2.71; p < 0.01).

**Figure 2:**
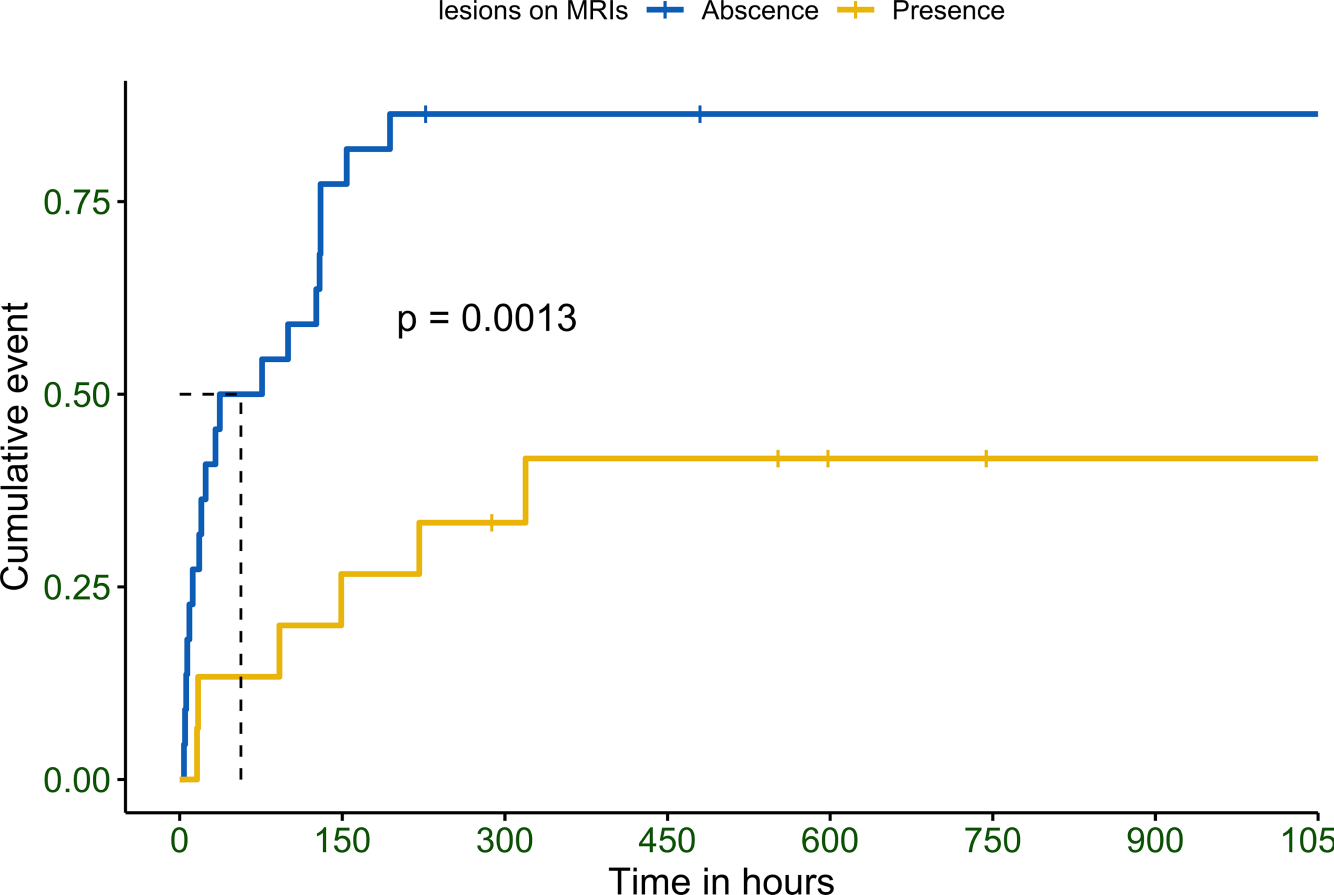
Observed Kaplan–Meier (KM) curve of probability for improvements in consciousness in patients with hypoglycemic encephalopathy depending on the detection of abnormal brain lesions in magnetic resonance imaging. P values calculated with the use of log-rank test.

Patients without lesions were found to recover much faster than those with lesions, regardless of the lesion location. Maximum time spent to recover was 194 in patients without brain lesions and 319 in those with lesions (**Figure 2**). None of the patients recovered consciousness after 320 h.

## Discussion

Our study is the first to report the time when treatment of HE becomes futile. To support this, we investigated prognostic factors, including MRI findings. This information is essential to the determination of the reversibility of HE and the therapeutic limitations of treatment. Our study showed that the absence of lesions with high signal intensity on diffusion-weighted imaging (DWI) was the most crucial factor in predicting improvement of consciousness in patients with decreased consciousness due to severe hypoglycemia. Furthermore, we confirmed that improvement of consciousness could occur even after 14 days after HE. Of note, majority of HE was developed in elderly with diabetes. In addition, all diabetic patients had been treated with insulin and/or sulfonylurea, which suggests that these agents should be cautiously prescribed in elderly.

Barbara et al. (6) reported similar results from the MRI scans of 20 patients with HE. Only 7% of their patients with poor outcomes showed normal initial MRI findings, whereas 25% of those with good outcomes had normal initial MRIs. In our study, 27.3% of patients had poor outcomes. In the study by Barbara et al., 63% had poor outcomes. This was despite more of our patients having severe gray matter lesions (n = 8, 53.3%) than those of Barbara et al. (35%). It is not fully established whether differences in lesion location are related to patient prognosis. However, some studies (9, 12) have reported that white matter is more vulnerable to hypoglycemia and has a greater association with irreversible injury. Therefore, white matter lesions during HE could appear early and result in irreversible damage. Yet, other studies have found that patients with gray matter involvement have worse outcomes than those with only white matter involvement (8, 13–15). Other research has demonstrated change over time in HE lesions with changes in blood glucose levels (12, 15–17). A prospective study (12) that performed DWI on admission and again on day 2 showed that gray matter lesions were more prominent on day 2 images than initial images, suggesting that these lesions had developed over time. In consecutive MRIs, we found lesions that moved from white to gray matter in some patients. One of these was a hyperintensity lesion to white matter and the hippocampus on admission. However, the follow-up image on day 12 showed extensive gray matter involvement without any lesion to the white matter. Another patient with only white matter involvement on admission showed extensive gray matter changes on a day 4 follow-up DWI, with regression of the white matter lesion. Both patients remained in a vegetative state. This suggests that lesion location might be affected by the time of the MRI study.

Previous studies have suggested that longer duration of hypoglycemia is a poor prognostic factor (6, 10). In our study, the duration of hypoglycemia was significantly shorter in patients who demonstrated GOS score improvements. However, the duration of hypoglycemia was not an independent predictor of improvement in GOS after correction for age, gender, initial consciousness, and presence of lesions on initial MRI. The definition of hypoglycemia duration, calculated as the time at which the first abnormality was detected, may have been somewhat arbitrary, however, and could have led to the underestimation of the effect of hypoglycemia duration. Meanwhile, studies have reported that profound hypoglycemia maybe a predictor for poor prognosis (10, 18). However, initial serum glucose concentration in our study was comparable in patients with or without consciousness improvement. This inconsistency might be caused by the difference in study population. Previous studies included patients with altered consciousness due to hypoglycemia regardless of recovery after glucose administration (10, 18). On the contrary, our study only included those who had persistent mental change after normalizing blood glucose levels. Thus, less profound hypoglycemia might be a factor related with early consciousness recovery after severe hypoglycemia, although it cannot predict the prognosis of prolonged HE.

Since prognostic evidence relating to severe HE is scarce, determination of the point at which it becomes irreversible is challenging. To date, no studies have investigated the time to recovery for HE. Barbara et al. reported that, in 37.3% of their HE patients, therapeutic limitations were determined using the same criteria as those for anoxic–hypoxic encephalopathy. They reported five cases of late recovery after ICU discharge and suggested at least 10–15 days before deciding on a treatment limit, even if there is no clinical improvement. Among previously reported cases, consciousness has been restored within 11 days (15) and 14 days (19). The latest improvements in consciousness observed in our study were at 319 h (13.3 days), even in patients with severe brain injury involving gray matter in two or more lobes. This indicates that decisions about the limitations of life-sustaining treatment should be taken very cautiously to avoid misdiagnosing the irreversibility of HE.

Our study had several limitations inherent to retrospective research. First, there were patients without follow-up MRI, and for those patients with these scans, the duration of follow-up imaging was different. Second, accurately assessing the onset of hypoglycemia was not easy because the information was based on medical records. We calculated the duration of hypoglycemia from the time at which the first abnormality was detected, but this may not have been accurate. Third, lactic acid levels were measured in few patients. Lactic acid has neuroprotective effects and low lactic acid level during hypoglycemia has been reported to be a possible poor prognostic (10). However, we could not evaluate the effect of lactic acid because the lactic acid levels were available in only a part of patients. Forth, we could not quantify alcohol consumption due to lack of data. It is well known that excessive alcohol consumption can cause brain damage through direct and indirect mechanism (20). Thus, history of heavy drinking not addressed in this study may inhibit neurological improvement of HE. Lastly, definition of consciousness improvement in this study might not be clinically meaningful because we also included those who had only a 1 point increase in GOS.

In conclusion, our study provides potentially helpful prognostic indicators of improvements in consciousness after HE and demonstrates that sufficient time is required to recover consciousness even in patients with severe brain damage. Further studies are needed to elucidate the location and mechanisms of brain lesions that cause changes in consciousness in HE. Follow-up data are also needed to avoid misdiagnosing irreversible HE.

## Data availability statement

The raw data supporting the conclusions of this article will be made available by the authors, without undue reservation.

## Ethics statement

The studies involving human participants were reviewed and approved by Dongguk University Ilsan Hospital Institutional Review Board. Written informed consent for participation was not required for this study in accordance with the national legislation and the institutional requirements.

## Author contributions

YEL contributed to the acquisition, analysis, and interpretation of data, and to drafting the manuscript. EJL contributed to the acquisition, analysis, and interpretation of data. SEL and JP contributed to the conception and design of the work, the acquisition, analysis, and interpretation of data and drafting of the manuscript. All authors contributed to the article and approved the submitted version.

## Funding

This work was supported by the Dongguk University Research Program 2021 and a grant from the National Research Foundation of Korea funded by the Korean Government (NRF- 2020R1C1C1009091) and the Korea Health Technology R&D Project through the Korea Health Industry Development Institute (KHIDI), funded by the Ministry of Health & Welfare, Republic of Korea (grant number: HI21C1074).

## Conflict of interest

The authors declare that the research was conducted in the absence of any commercial or financial relationships that could be construed as a potential conflict of interest.

## Publisher’s note

All claims expressed in this article are solely those of the authors and do not necessarily represent those of their affiliated organizations, or those of the publisher, the editors and the reviewers. Any product that may be evaluated in this article, or claim that may be made by its manufacturer, is not guaranteed or endorsed by the publisher.
